# Right procedure, wrong organ, an unusual case report of aortic trauma in a multiple injured patient

**DOI:** 10.4076/1757-1626-2-6795

**Published:** 2009-06-05

**Authors:** Aristotelis P Mitsos, Jonathan Chantler, Evangelos Konstantinou, Theofanis Fotis, Ekaterini Lambrinou, Ramon Uberoi, Richard Stacey, James V Byrne

**Affiliations:** 1 Department of Neuroradiology, West Wing, John Radcliffe HospitalHeadley Way, Headington, Oxford, OX3 9DUUK; 2 Department of Anaesthesiology, West Wing, John Radcliffe HospitalHeadley Way, Headington, Oxford, OX3 9DUUK; 3 Evgenidion University Hospital Department of Nurse Anaesthesiology, National and Kapodistrian University of Athens, Faculty of NursingAthensGreece; 4 Vascular Surgery Department, 251 Hellenic Air Force Hospital; 5 Nursing Department, School of Health Sciences, Cyprus University of Technology; 6 Department of Cardiology and Neurosurgery West Wing, John Radcliffe HospitalHeadley Way, Headington, Oxford, OX3 9DUUK; 7 Department of Cardiology and Neurosurgery West Wing, John Radcliffe HospitalHeadley Way, Headington, Oxford, OX3 9DUUK

## Abstract

Blunt traumatic injury and acute dissection of thoracic aorta is increasing in incidence in seriously multi-trauma patients, remaining highly lethal. Early identification and repair is the key to a successful outcome. We report an unusual case of a 62-year-old man involved in a motor vehicle accident after subarachnoid hemorrhage due to an intracranial artery aneurysm rupture. The post-traumatic aorta dissection was overlooked during the initial evaluation and was found incidentally later during an attempt for endovascular treatment of the intracranial aneurysm. The pitfalls in the diagnostic approach of this patient are discussed and the paramount importance of the correct interpretation of all the available clinical and investigational findings in multiple injured patients are highlighted.

## Introduction

Multiply-injured patients are always a diagnostic and therapeutic clinical challenge. Only a very careful study and interpretation of the trauma history and conditions, the clinical presentation and examination and the appropriate radiological investigation will identify all the related injuries [1]. Such interpretation will offer the key to the correct diagnosis and will also dictate the priorities in the treatment strategy.

## Case presentation

**Figure 1.**
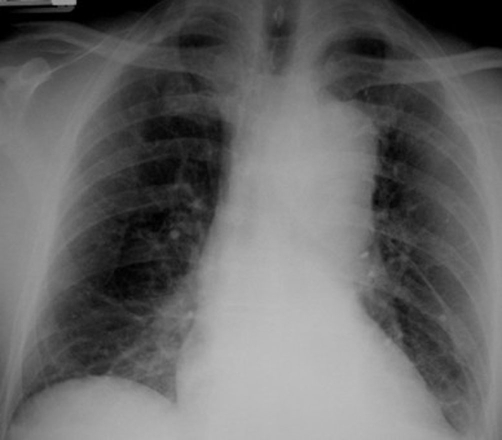
AP chest X-ray on admission.

**Figure 2.**
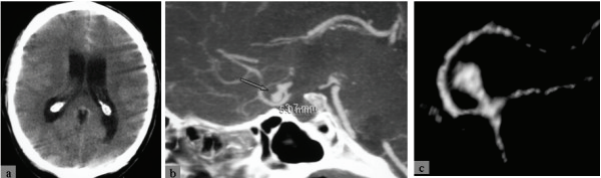
Brain CT scan on admission **(A)**. CT angiogram revealed an anterior communicating aneurysm measuring 3.7 x 5 mm **(B arrow, C)**.

**Figure 3.**
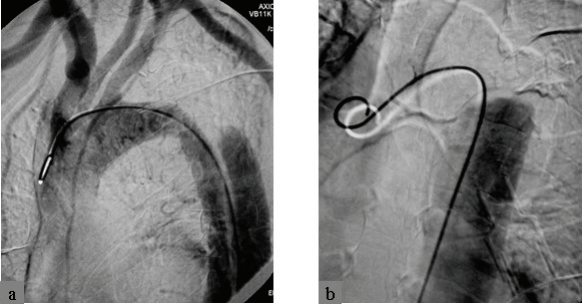
Aortogram showing the dissected segment of the descending aorta distal to the origin of the left subclavian artery **(A)**. Note the retention of the contrast medium in the dissected segment of the artery **(B)**.

**Figure 4.**
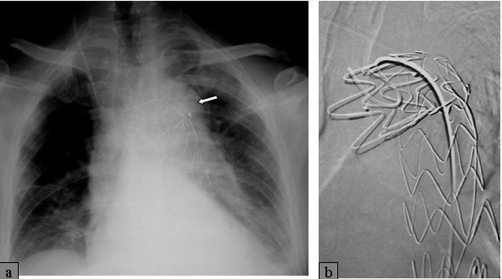
Chest X-ray two weeks after successful aorta stent deployment.

**Figure 5.**
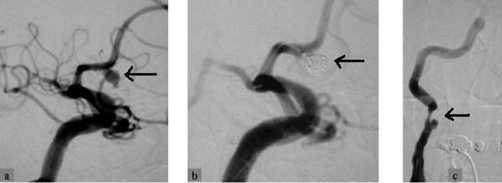
Two weeks later, the Acom aneurysm, was endovascularly treated with platinum coils **(A, B)**. Traumatic dissection of the Rt cervical ICA **(C)** was treated conservatively at that stage.

A 62-year-old, white, Caucasian, British-origin, male lorry driver was admitted to the hospital late on a Friday night after an unwitnessed single vehicle road accident. His lorry was by the side of the road and he was found unconscious in the vehicle and immediately transferred to the local District General Hospital. On admission, he had already fully recovered, being alert and orientated but he had no recollection of the accident. He was haemodynamically stable, with normal vital signs and no neurological deficit. He complained only at a moderate headache. Physical examination has revealed a bruise on the upper part of his left anterior chest wall, left shoulder and forehead. The rest of the examination was entirely normal. The radiological investigation that followed included cervical spine and left arm X-rays in which no fracture was noted. A mobile AP chest X-ray was also done on admission, in which it was demonstrated a dilated aortic arch (Figure 1). A subarachnoid hemorrhage was found on a moderate quality brain CT-scan (Figure 2A), which was attributed to traumatic brain injury and the patient was transferred to the regional Neurosurgical centre as a post-traumatic subarachnoid hemorrhage for observation. During the next two days, the patient was well without any neurological defect and he has been gradually mobilized without any major complaint. On Monday however, brain CT interpretation by a more senior member of the medical staff suggested that this may not be a post-traumatic but rather an aneurismal subarachnoid hemorrhage (SAH). A CT angiogram confirmed that his SAH was of aneurismal origin, due to the rupture of an anterior communicating artery (Acom) aneurysm (Figure 2B) which was the actual reason for his accident. As the patient was neurologically intact and stable, an embolisation procedure for endovascular treatment of this aneurysm was decided for the following day (day 5 since the SAH event). Under general anesthesia, the right common femoral artery was punctured and a 6F appropriate vascular sheath was placed following our protocol for endovascular procedures. During the first attempt to catheterize the aortic arch for the standard pre-embolisation angiogram, an unusual dilatation of the thoracic descending aorta was noted, just distal to the origin of the left subclavian artery. The combination of these findings with the bruise in the upper left chest, made us suspicious of an aortic injury. The embolisation procedure was abandoned and an aortogram revealed an aortic dissection just distal to the left subclavian origin (Figure 3). The patient was woken up and a chest CT followed, which confirmed the diagnosis of a post-traumatic dissection of the descending aorta. Subsequently, he was transferred to the cardio-thoracic unit and an aortic stent was successfully deployed for endovascular repair of the dissection (Figure 4). He had an uneventful recovery and two weeks later, endovascular treatment of his Acom aneurysm was performed with platinum coils (Figure 5A,B). At the same time, a posttraumatic dissection aneurysm of the right common carotid artery (CCA) at its cervical segment was also found (Figure 5C). This was clinically silent, treated conservatively at that stage [3] and the patient was eventually discharged three weeks after the primary event. At the six month follow-up, the patient had made a very good recovery and returned to full activities. The aortic dissection was not visible anymore in imaging but there was a recurrence of the previous coiled Acom aneurysm due to coil compaction. Thus, a new embolisation procedure with platinum coils was performed to treat this aneurysm recurrence. At the same stage, the right CCA dissection aneurysm was endovascularly treated using platinum coils, as it showed no signs of regression [2,3].

## Discussion

Acute traumatic injury of the thoracic aorta is a relatively common injury of deceleration accidents, usually high-speed motor vehicle accidents [3] that still remains highly lethal [1,4]. As a consequence of major car crashes, thoracic aortic ruptures occur in 10 to 30% of multiply injured patients [5]. They represent the second most common cause of death after trauma, following only the head injuries [6]. Only 15% of these patients reach the hospital alive and their management is further complicated by other lesions in the skeletal and visceral organs with significant difficulties in their diagnosis [7]. Frequently, these patients have also multiple other severe injuries [1,8,9] which may draw the main attention of the involved physicians in an Accident and Emergency unit, causing a delay in the diagnosis and treatment of post-traumatic aortic dissections. Early diagnosis of dissection is difficult as it mainly relies on high index of suspicion based on the mechanism of the injury. Furthermore, clinical signs are not sensitive or specific and chest X-rays may not identify the problem in a high number of multiply injured patients [10]. Before hospital admission, the primary cause of death is hemorrhage, and only a small percentage of patients who actually receive medical attention survive [11,12]. The most important factor to successful hospital management of these victims is early diagnosis by using multislice CT and appropriate urgent treatment that may lead up to 80% of survival [8,13]. In our case, the situation was even more complicated. The cause of the road accident and subsequent patient injury was an episode of subarachnoid hemorrhage due to the rupture of a cerebral aneurysm. As the brain CT was misdiagnosed, a supposed brain injury attracted the main attention and the co-existent aortic dissection was undetected. When the correct diagnosis of aneurismal subarachnoid hemorrhage was made, the interest was even more concentrated on the possible brain lesion, which also required an urgent treatment. It is well known that a ruptured intracranial aneurysm has a high risk of re-bleeding; this risk is higher immediately after the first event (30% during the first three weeks) carrying also an extremely high mortality rate (50%) [14]. The aorta is the largest arterial vessel of the body and injuries that may lead to its rupture (i.e. aortic dissection) are potentially life threatening [1,12,8,13,15]. Aortic dissections are usually classified - for treatment purposes - as those involving the ascending aorta (i.e. type A dissections), which are usually managed surgically [6] and type B traumatic dissections which are usually managed with endoluminal aortic stents [9,16]. Traumatic aortic rupture commonly follows an anterio-posterior thoracic injury, with 60% occurring just distal to the origin of the left subclavian artery (as in our case) and less frequently (25%) at the ascending aorta [15]. Treatment options in these high risk injuries include either open surgical or endovascular repair [3]. Endovascular treatment of thoracic aortic diseases, even in the acute phase, may represent an alternative valid option with a low mortality rate and an excellent outcome [8,9,13,17]. This has been also proved in our case. Following the repair of the aortic dissection, it was then safe to secure the intracranial aneurysm by endovascular treatment with platinum coils. Two main pitfalls can be noted in the management of this patient. Both of them have to do with misinterpretation of the patient history, clinical examination and radiological investigation. The *first* was the misdiagnosis of posttraumatic instead of aneurismal subarachnoid hemorrhage, which was the actual cause of the accident. This was due to the inability of the patient to describe the event due to retrograde posttraumatic amnesia as well as due to the inexperience of the admitting physician to identify the hinds of the brain CT scan indicating the aneurismal origin of the subarachnoid hemorrhage. The *second* was the underestimation of the chest injury, regarding either the clinical signs or the chest X-rays. It is reported that 65% of chest trauma in multiple injured patients may be overlooked in plain chest X-rays [18]. Similarly in our case, retrospective review of the patient's chest X-rays identified widened superior mediastinum as well as descending aorta, both findings highly suspicious of an aortic dissection, especially in relation to the clinical finding of chest bruise [19].

## Conclusion

The present case report underlines the major importance of the correct interpretation of all the available information and data (trauma history, physical examination, radiological investigation) regarding multiple-injured patients. A systemic process to this particular type of patients is extremely valuable. Early diagnosis of an aortic dissection in a multiply injured patient is often difficult but also particularly important for the final outcome. The early identification of all the possible injuries and their thorough clinical and radiological investigation are of paramount importance for an effective treatment approach and a favorable clinical outcome.

## List of abbreviations

CCA: Common carotid artery
CT: Computed tomography
SAH: Subarachnoid hemorrhage
